# Gut microbial profile of treatment-naive patients with primary biliary cholangitis

**DOI:** 10.3389/fimmu.2023.1126117

**Published:** 2023-05-08

**Authors:** Yi-jun Zhou, Gao-xiang Ying, Shi-lei Dong, Bo Xiang, Qiao-fei Jin

**Affiliations:** ^1^ Department of Hepatology, Affiliated Hangzhou Xixi Hospital Zhejiang University School of Medicine, Hangzhou, Zhejiang, China; ^2^ Department of Clinical Laboratory, Zhejiang Hospital, Hangzhou, Zhejiang, China

**Keywords:** primary biliary cholangitis, fecal microbiome, biomarkers, amplicon sequence variants (ASV), diagnosis

## Abstract

**Background and aims:**

The pathogenesis of primary biliary cholangitis (PBC) is associated with alterations of gut microbiota. We compared the gut microbiota of PBC patients and healthy controls from Zhejiang Province and assessed the use of these data for the diagnosis of PBC.

**Methods:**

First, 16S rRNA gene sequencing was used to characterize the gut microbiota of treatment-naive PBC patients (n=25) and matched healthy controls (n=25). Then, the value of gut microbiota composition for the diagnosis of PBC and assessment of PBC severity was determined.

**Results:**

The gut microbiota of PBC patients had lower diversity based on three different metrics of alpha-diversity (ace, Chao1, and observed features) and fewer overall genera (all p<0.01). PBC patients had significant enrichment of four genera and significant depletion of eight genera. We identified six amplicon sequence variants (*Serratia*, *Oscillospirales*, *Ruminococcaceae*, *Faecalibacterium*, *Sutterellaceae*, and *Coprococcus*) as optimal biomarkers to distinguish PBC patients from controls based on receiver operating characteristic analysis (area under the curve [AUC] = 0.824). PBC patients who were anti-gp210-positive had lower levels of *Oscillospiraceae* than those who were anti-gp210-negative. KEGG functional annotation suggested the major changes in the gut microbiota of PBC patients were related to lipid metabolism and biosynthesis of secondary metabolites.

**Conclusion:**

We characterized the gut microbiota of treatment-naive PBC patients and healthy controls from Zhejiang Province. The PBC patients had significant alterations in their gut microbiota, suggesting that gut microbiota composition could be useful as a non-invasive tool for the diagnosis of PBC.

## Introduction

Primary biliary cholangitis (PBC) is a chronic auto-immune cholestatic liver disease that is characterized by fibrosis and destruction of the interlobular bile ducts (1). Studies of the pathogenesis of PBC have mainly focused on genetic susceptibility (2), environmental factors (3), and immune factors (4). However, there is increasing emphasis on the effect of gut microbiota in PBC (5, 6) because the liver and intestine are linked by the portal vein, forming a gut-liver axis (7). Previous studies confirmed that many patients with chronic liver diseases, including PBC, have different severities of dysbiosis of gut microbiota (8). Disruption of the tight junctions (TJs), important structures composed of multiple proteins that help to maintain intestinal homeostasis, can lead to a leaky gut (9). Patients with PBC often have many gut microbes and metabolites that can penetrate the intestinal mucosal barrier and are then transported to the liver *via* the gut-liver axis (10), where they can trigger an immune-mediated attack against the small bile duct (11). Therefore, studies of alterations in the gut microbiota of patients with PBC may improve the diagnosis and treatment of this disease and understanding of its pathogenesis.

Few previous studies have examined the characteristics of the gut microbiota of PBC patients, although there were two notable recent studies of this topic. Furukawa et al. (12) studied PBC patients from Japan and reported they had significantly reduced diversity of gut microbiota, with abnormal increases of *Enterococcus*, *Streptococcus*, *Lactobacillus*, and *Bifidobacterium*, and a significant decrease of *Clostridiales*. They also found that the use of ursodeoxycholic acid (UDCA) and proton pump inhibitors (PPIs) were important confounding factors because they affected the composition of gut microbiota. Tang et al. (13) studied PBC patients from the outpatient clinic of Shanghai Renji Hospital and found alterations in their fecal flora, with increases in eight bacterial genera and decreases in four bacterial genera relative to controls. In particular, *Klebsiella* was significantly more abundant in PBC patients and positively correlated with the level of serum total bilirubin. However, there have been no studies of the gut microbiota of patients with PBC in Zhejiang Province, and populations from different regions often have significant differences in their gut microbiota (5).

In this study, we compared the characteristics of the gut microbiota in treatment-naive PBC patients and healthy controls from Zhejiang Province to identify specific gut microbiota markers that have potential use for the diagnosis and treatment of PBC. Our focus was to determine the correlation of different gut microbiota with liver functional indexes, and then evaluate the use of gut microbiota for the diagnosis of PBC and determination of PBC severity in an effort to reduce the need for invasive testing.

## Methods

### Study cohort and sample collection

This study compared 25 treatment-naive PBC patients and 25 healthy matched controls. All PBC patients were from the inpatient department of Hangzhou Xixi Hospital affiliated to Medical School of Zhejiang University, and healthy controls were from the physical examination center. Blood samples were collected for analysis of liver function (including alkaline phosphatase [ALP], gamma-glutamyl transferase [GGT], alanine transaminase [ALT], aspartate transaminase [AST], and total bilirubin [TB]) and immunological tests (including IgG, IgM, anti-mitochondrial antibodies [AMA], anti-mitochondrial M2 antibodies [AMA-M2], anti-sp100 antibodies, and anti-gp210 antibodies). Fecal samples were collected for analysis of gut microbiota. Each PBC patient received an ultrasound examination and a liver biopsy. All fecal samples were freshly collected at the hospital, stored using the Longseegen Stool Storage Kit (No : LS-R-P-007, Guangdong, China), and frozen at −20°C within 3 h after collection. Histological analyses of liver samples were evaluated using the Ludwig staging system (14). All samples were collected from June 2021 to June 2022.

The 2018 PBC practice guidelines of American Association for the Study of Liver Diseases (AASLD) (13) were used for the diagnosis of PBC. Each enrolled PBC patient had all three of the following criteria: (*i*) elevation of ALP; (*ii*) presence of AMAs or other PBC-specific auto-antibodies (including those against sp100 or gp210); and (*iii*) histology results indicating nonsuppurative destructive cholangitis with destruction of the interlobular bile ducts. The two major exclusion criteria for PBC patients were: (*i*) previous standardized treatment for PBC or use of UDCA and (*ii*) use of antibiotics, lactulose, probiotics, PPIs or other drugs that might alter the gut microbiota within the previous 2 months. Patients with PBC-autoimmune hepatitis (PBC) overlap syndrome, as defined by the Paris criteria (15), were also excluded. All healthy controls, who were matched for age, sex, and BMI, had the following characteristics: (*i*) no significant abnormalities in routine blood tests, liver or kidney function tests, fasting glucose, serum lipids, or liver ultrasound results; (*ii*) no infection by the hepatitis B or C virus; and (*iii*) no use of antibiotics, probiotics, or other drugs that might alter gut microbiota within the previous 2 months.

This study was conducted according to the principles of the Declaration of Helsinki and was approved by the Medical Ethics Committee of Hangzhou Xixi Hospital (Ethical Approval No. 2020, Science and Education Section, Medical Ethics Committee No. 41). Each study participant signed a written informed consent document before participation.

### DNA extraction and 16S ribosomal RNA gene sequencing

Primers were designed to amplify specific regions of the 16S V3-V4 region, and an amplified fragment of about 420 bp was obtained. The paired-end data of 2×250 bp were sequenced using the Illumina Novaseq 6000 platform, and longer sequences were obtained by splicing for 16S analysis. Standard data cleansing techniques were used to improve the accuracy and reliability of these measurements. In particular, the raw sequencing data were first de-noised and filtered to obtain validated (cleansed) data, and clustering of amplicon sequence variants (ASVs) and species classification were then performed using the validated data. Based on the clustering results of the ASVs, taxon identification and abundance were determined, with annotation for each ASV sequence. ASVs were also analyzed to determine species richness and evenness within samples. Common and unique ASVs among different samples or groups were identified by determining their abundance, and were presented in Venn diagrams, petal diagrams, and calculations of alpha diversity. Data obtained from the National Center for Biotechnology Information (NCBI) 16S rRNA database (BioProject ID : PRJNA892581, https://submit.ncbi.nlm.nih.gov/subs/bioproject/SUB12178067/overview) were used for identification.

### Bioinformatic analysis of 16S rRNA sequencing

PICRUSt (https://picrust.github.io/picrust/) is a bioinformatics tool that uses 16S rRNA sequences to determine the functional profiles of microbial communities (16). Determination of the gene functions of sequenced microbial genomes also allows comparisons of different groups. Functional gene abundance enrichment of the Kyoto Encyclopedia of Genes and Genomes (KEGG) pathways at different levels (1–3) were obtained using PICRUSt functional prediction from the 16S rDNA sequences. Clusters of Orthologous Genes (COG), KEGG Orthology (KO), and KEGG metabolic pathway predictions were also performed.

### Statistical analysis

SPSS version 22.0 (IBM Corp., Armonk, NY, USA) was used for statistical analysis and calculation of the statistical significance of differences between groups. Linear discriminant analysis effect size (LEfSe) analysis was used to identify differences in taxa and pathways between the two groups. Receiver operating characteristic (ROC) curves were constructed and area under curve (AUC) values were calculated to assess the diagnostic performance of the model using the pROC package in R software. Spearman’s rank correlation was calculated to determine correlations between the two groups, and Pearson’s correlation was calculated to determine correlations of gut microbiota with clinical indexes. A Wilcoxon rank-sum test was used to determine the significance of differences in continuous variables in the two groups.

## Results

### Characteristics of participants

We used strict patient selection and exclusion criteria, and collected stool samples from 25 treatment-naive PBC patients and from 25 healthy controls, with matching for age, gender, and BMI (**Table 1**). Most of the PBC patients were middle-aged women, and the two groups were similar in most baseline measurements except GGT and ALP, which were higher in the PBC patients (both P < 0.001). Measurements of autoantibodies showed that 96% of PBC patients were positive for AMA-M2, 60% were positive for anti-sp-100 antibodies, and 84% were positive for anti- gp210 antibodies. The ultrasound examinations of all PBC patients indicated no obvious abnormalities, such as signs of a liver mass or cirrhosis. Liver biopsy testing showed that all PBC patients had Ludwig stage I or II disease.

**Table 1:** Characteristics of treatment-naive PBC patients and matched healthy controls.

Characteristic	PBC patients (n=25)	Controls* (n=25)	*p* value
Age, median years (min–max)	55 (43–77)	57 (24–77)	0.979
Female, n (%)	21 (84%)	22 (88%)	0.946
BMI, median kg/m^2^ (min–max)	21.33 (19.12–23.5)	20.77 (20.12–21.56)	0.058
Hepatic function tests, median (min–max)			
ALP, U/L	199 (144–392)	77 (60-100)	0.001#
GGT, U/L	150 (103–292)	32 (21-45)	0.001#
ALT, U/L	25 (15–45)	25 (16-40)	0.345
AST, U/L	33 (17–69)	24 (16-50)	0.161
TB, μmol/L	14.6 (4.79–45.17)	17.1 (13.9-28.8)	0.865
Immunoglobulins, median (min-max)			
IgG, g/L	14.05 (9.37–31.66)		
IgM, g/L	2.62 (0.94–9.13)		
Autoantibody positivity, n (%)			
AMA	20 (80%)		
AMA-M2	24 (96%)		
sp100	15 (60%)		
gp210	21 (84%)		
Histological results			
Ludwig stage I/II/III/IV, n (%)	16/9/0/0 (64%, 36%, 0%, 0%)		

*Data from controls were from routine physical examinations, and therefore did not include immunoglobulins, autoantibodies, and histological results. # with staitistical significance.

### Fecal microbiomes in PBC patients and controls

**Figure 1:**
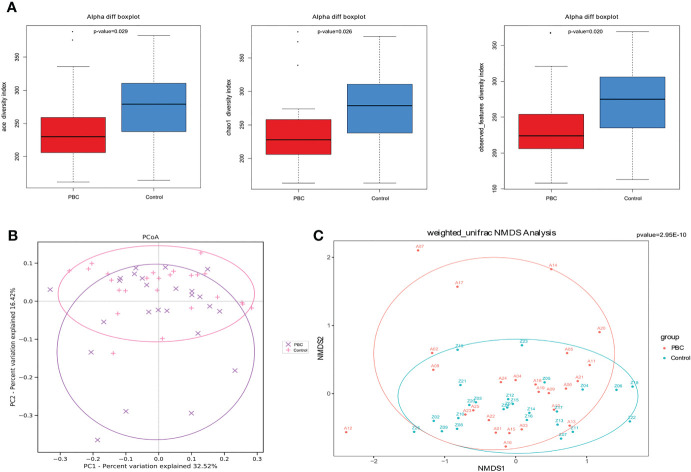
Gut microbial diversity in PBC patients and healthy controls. **(A)** Ace diversity index (left), Chao1 diversity index (center), and observed features index (right) were significantly lower in PBC patients. **(B)** PCoA based on weighted UniFrac matrix analysis showed the two groups had differences in overall fecal microbiota composition (p < 0.01). **(C)** Beta-diversity based on was NMDS analysis of the weighted UniFrac matrix showed distinct separation of the two groups in the direction of the NMDS2 axis (p<0.01).

**Figure 2:**
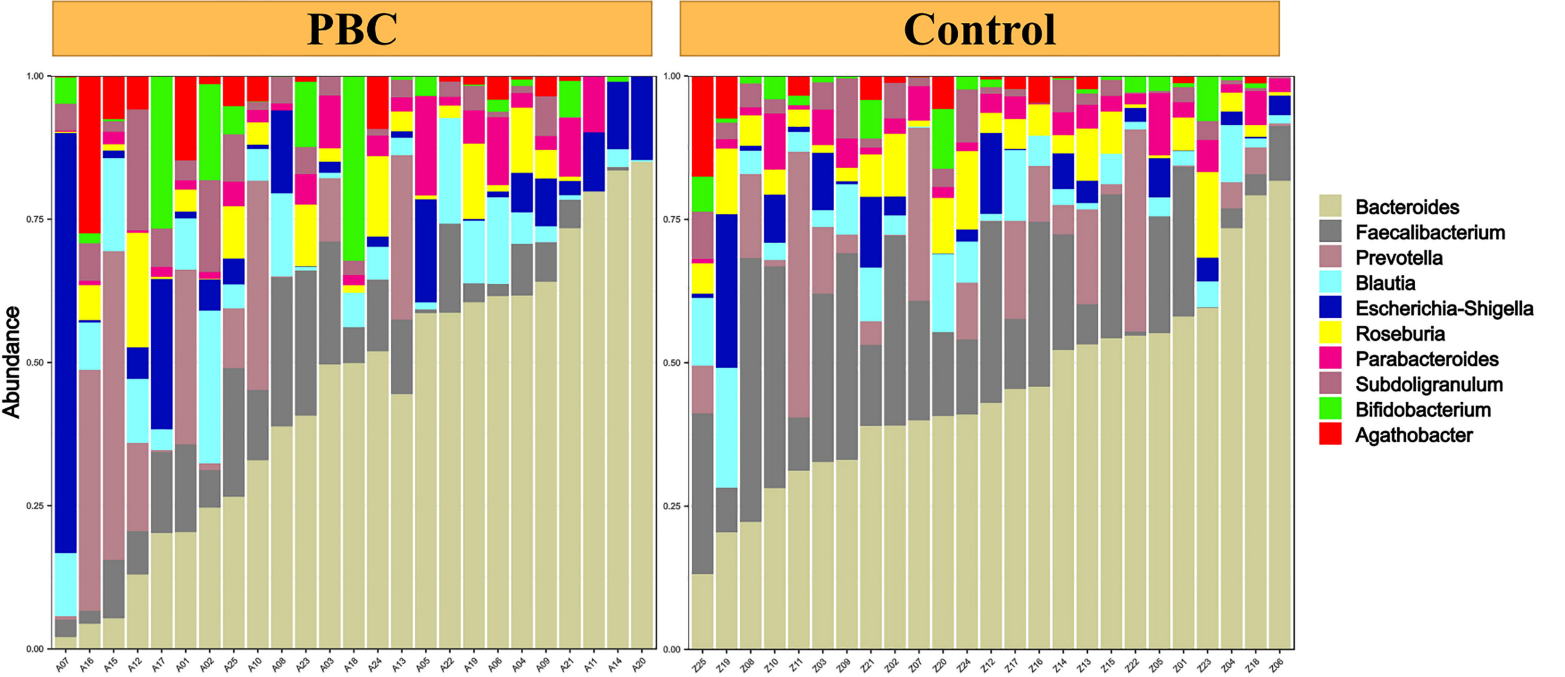
Abundance of different taxa in the fecal microbiota of each PBC patient (left) and each healthy control (right).

Analysis of alpha diversity of the microbiomes of PBC patients and controls showed that the PBC patients had significantly reduced richness and evenness (both P < 0.05, **Figure 1A**). We also compared the two groups in terms of beta-diversity using principal coordinate analysis (PCoA) (**Figure 1B**) and non-metric multidimensional scaling with 2 axes (NMDS2) (**Figure 1C**). These results also indicated significant intergroup differences. Therefore, the fecal microbial communities in patients with PBC were distinct from those of the healthy controls. Consistent with these results, a comparison of the overall composition of the fecal microbiomes in the two groups indicated many taxonomical differences (**Figure 2**).

### Phylogenetic characteristics of the fecal microbial communities in PBC patients

**Figure 3:**
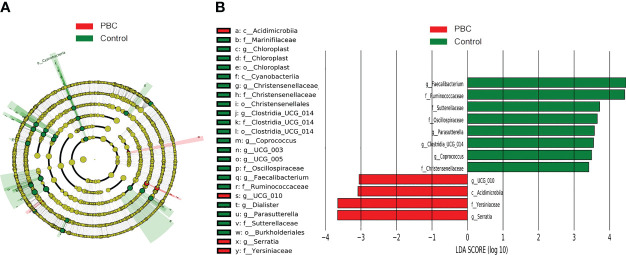
LEfSe and LDA analyses based on ASV characterizations of microbiota in PBC patients and matched healthy controls. **(A)** Cladogram. Red: PBC group, Green: control group, Node color: microbial groups that play important roles, yellow node: microbial groups that do not play an important role. Gut taxa indicated by the letters in the figure are shown in the legend on the right. **(B)** Twelve gut taxa with LDA scores greater than 3 or less than −3. Green: enriched in the control group, Red: enriched in the PBC group.

LEfSe cladogram analysis of PBC patients (red) and controls (green) indicated clear differences between these groups (**Figure 3A**). Least discriminant analysis (LDA) of genus scores showed that 12 microbial biomarkers clearly distinguished PBC patients and controls (**Figure 3B**). In particular, there were 4 predominant genera in PBC patients (*Acidimicrobiia*, *Yersiniaceae, Serratia*, and *ucg_010*; all P < 0.05 and LDA > 3), and 8 predominant genera in the controls (*Faecalibacterium*, *Ruminococcaceae*, *Sutterellaceae*, *Oscillospiraceae, Parasutterella, Clostridia, Coprococcus*, and *Christensenellaceae*; all P < 0.05 and LDA > 3).

### Gut microbiome signature and diagnosis of PCB

**Figure 4:**
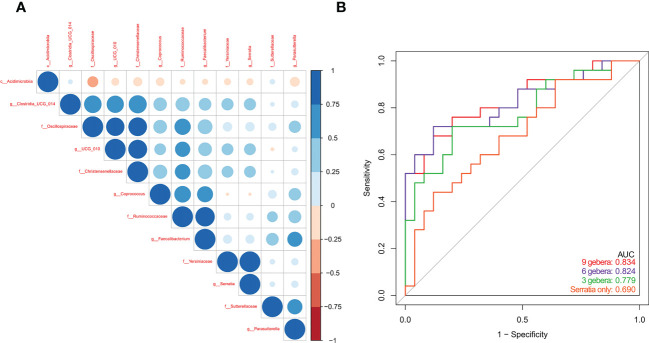
**(A)** Spearman correlations of the 12 PBC-associated genera in treatment-naive PBC patients and healthy matched controls. **(B)** ROC analysis based on a multivariable logistic regression model using 9 genera, 6 genera, 3 genera, and Serratia alone.

Spearman correlation analysis of the 12 microbial genera identified above showed negative correlations in the abundances of genera enriched in PBC patients with genera enriched in controls (**Figure 4A**). Multivariable stepwise logistic regression analysis showed that 6 genera (*Serratia*, *Oscillospirales*, *Ruminococcaceae*, *Faecalibacterium*, *Sutterellaceae*, and *Coprococcus*) reliably discriminated PBC patients from controls (data not shown). Subsequent ROC analysis based on these 6 genera led to an AUC of 0.824 (95% CI: 0.71, 0.94, **Figure 4B**). ROC analysis using 9 genera did not significantly improve the predictive performance (AUC: 0.834, 95% CI: 0.72, 0.95).

### Correlation of fecal microbiome characteristics and PBC disease severity

**Figure 5:**
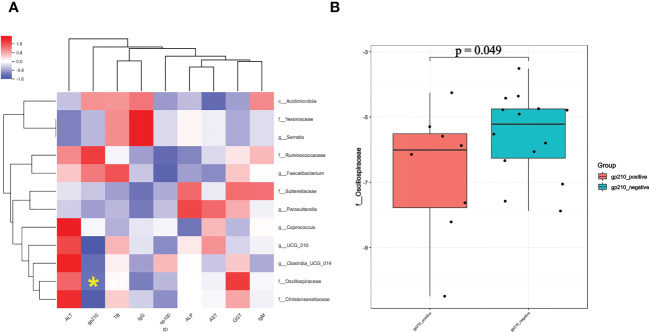
**(A)** Pearson correlation coefficients for the relationships of 12 gut genera (vertical axis) with 9 clinical indices (horizontal axis) in PBC patients (red: positive correlation, blue: negative correlation). **(B)** Level of Oscillospiraceae in PBC patients who were anti-gp-210 positive and anti-gp-210 negative. *enrichment of Oscillospiraceae was negatively related to anti-gp210 antibody status.

We then used Pearson’s rank test to analyze the relationship of PBC-associated genera and clinical indices of disease severity in PBC patients, with control for potential interference by age, gender, and BMI. The results showed that enrichment of *Serratia* and *Yersiniaceae* were positively related to the IgG level, and enrichment of *Oscillospiraceae* was negatively related to anti-gp210 antibody status (**Figure 5A**). In addition, calculation of the level of *Oscillospiraceae* in patients who were anti-gp210-positive and anti-gp210-negative showed that the anti-gp210- positive group had a lower level of *Oscillospiraceae* (P < 0.05, **Figure 5B**).

**Figure 6:**
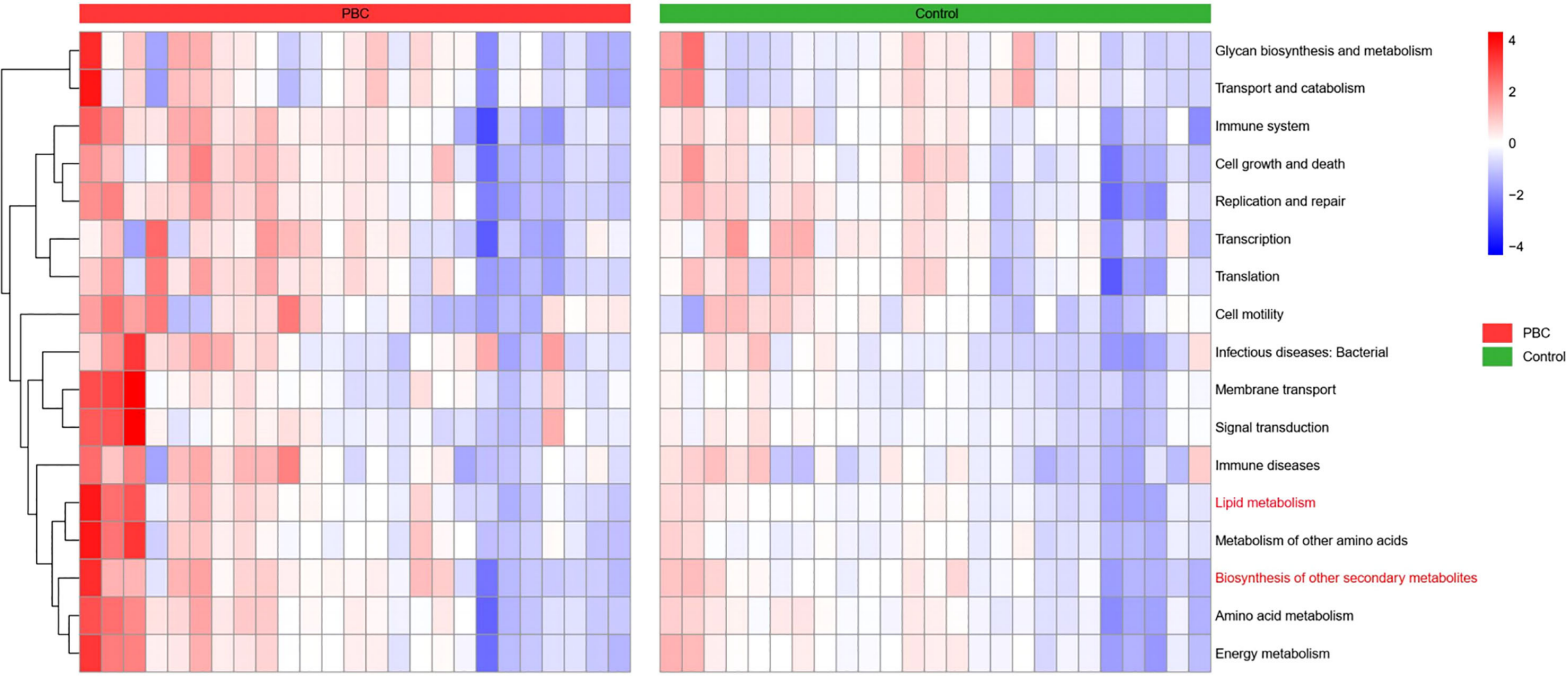
Heatmap of Spearman correlations between KEGG categories (vertical axis) and the levels of microbiome taxa (horizontial axis) in the PBC group (left) and the control group (right).

We then compared the functional and metabolic profiles of the gut microbial communities in PBC patients and controls using PICRUSt (**Figure 6**). The results indicated that 42 KEGG categories were significantly different in PBC patients and controls. Notably, the categories of lipid metabolism and biosynthesis of other secondary metabolites were abnormal in the microbiomes of PBC patients.

## Discussion

We used 16s rRNA sequencing of gut microbiota to compare 25 treatment-naive PBC patients and 25 matched controls who were from Zhejiang Province. Diversity measures indicated the PBC patients had significantly reduced richness and evenness of gut microbiota. Our NMDS2 and PCoA results also indicated significant differences in the abundances of different gut microbiota in PBC patients and controls. Our differential gut microbiota analysis of the 2 groups identified significantly greater abundances of 4 taxa and significantly reduced abundances of 8 taxa in PBC patients. Spearman correlation analysis showed a negative correlation in the abundance of *Acidimicrobiia* and *Oscillospiraceae*; enrichment of *Oscillospiraceae* and *Christensenellaceae* in the controls; and enrichment of *Serratia* and *Yersiniaceae* in the PBC patients. Multivariable logistic regression indicated that 6 ASVs (*Serratia*, *Oscillospirales*, *Ruminococcaceae*, *Faecalibacterium*, *Sutterellaceae*, and *Coprococcus*) were optimal biomarkers for distinguishing PBC patients from matched controls. Subsequent ROC analysis indicated these taxa provided an AUC of 0.824, and the AUC value of *Serratia* alone was 0.690.

Correlation analysis of different clinical indices with the 12 taxa that differed between the groups indicated that PBC patients who were anti-gp210-positive had lower levels of *Oscillospiraceae* than patients who were anti-gp210-negative. Previous research found that anti-gp210-positive PBC patients tend to have a poorer prognosis and to develop a range of complications, especially earlier onset of portal hypertension (17). Therefore, we speculate that a severe depletion of *Oscillospiraceae* may be a predictor of poor prognosis. In addition, the serum IgG level in PBC patients was positively correlated with the levels of *Serratia* and *Yersiniaceae*. Although elevated serum IgM levels are more common in PBC patients than in patients with other autoimmune liver diseases (18), some PBC patients with systemic rheumatic diseases (especially Sjögren’s syndrome) have elevated serum IgG levels (19). Therefore, we speculate that if a PBC patient presents with abnormally enriched *Serratia* and *Yersiniaceae* in the gut microbiota, clinicians should remain alert to the possibility of other extrahepatic autoimmune diseases.

Other studies of the gut microbiota in PBC patients examined patients from different geographical locations. A study in China reported sequences from the gut microbiota of 394 healthy subjects from seven different cities and found that ethnicity and especially geographic location were the main factors affecting gut microbiota composition (20). The most enriched genus in our PBC patients was *Serratia*, but a study performed in Shanghai reported the most enriched genus in PBC patients was *Klebsiella (* 2), and a study in Japan reported that *Lactobacillales* was the most enriched genus (12), although all of these genera are in the *Enterobacteriaceae*. A recent study in Shenzhen (southern China) showed that the normal bilirubin group of PBC patients had a lower abundance of *Gemmiger*, *Blautia*, *Anaerostipes*, and *Coprococcus* genera than a high bilirubin group of PBC, in which *Holdemania* was absent (21).Therefore, when screening gut flora for the diagnosis of PBC it is important to consider the geographical location of the patient.

PBC patients are diagnosed using a combination of biochemical markers, autoantibodies, and liver histopathology. Although most guidelines do not require liver histopathology for diagnosis, all PBC patients in our study received liver biopsies and assessment using the Ludwig staging system. All of our patients had Ludwig Stage I or II. There were several reasons for our focus on patients with early-stage PBC. First, the main aim of this study was to assess the value of gut microbiota composition as an early diagnostic marker for PBC. Second, patients with advanced-stage PBC are often hospitalized for various complications, especially due to portal hypertension, and often receive medications that can alter the gut microbiota. All of our PBC patients were treatment-naive and had no history of using UDCA. UDCA can reshape the bile acid profile (22) and affect the gut microbiota composition.

Our results indicated that PBC patients had 4 significantly up-regulated genera and 8 significantly down-regulated genera. Three microbiota alterations particularly attracted our attention.

First, *Serratia* and *Yersiniaceae* were significantly enriched in PBC patients. When there is an intestinal microecological imbalance, an immune response against bacterial antigens may also lead to attacks of structurally similar human antigens — an autoimmune-mediated injury (23). There is evidence that antibodies from patients with autoimmune liver diseases react with specific microbial proteins. For example, the AMAs from PBC patients bind to *Escherichia coli* proteins (24), and *Serratia*, *Yersiniaceae*, and *E. coli* are all in the *Enterobacterales*. Therefore, we speculate that a “mosaic effect” occurs (25)when the body creates antibodies against *Serratia*, *Yersiniaceae*, and their metabolites and these antibodies then mistakenly attack bile duct epithelial cells, manifesting as immune-mediated bile duct injury.

A second notable alterations is that the level of *Faecalibacterium* was significantly reduced in PBC patients, a finding apparently unique to PBC patients from Zhejiang Province. *Faecalibacterium* is a major producer of intestinal butyrate and plays a crucial role in maintaining intestinal homeostasis and host health (26). Butyrate is one of the main energy sources of colon cells, and is important for maintaining the integrity of the intestinal mucosal barrier (27). More specifically, butyrate strengthens the intestinal mucosal barrier by activating AMP-activated protein kinase (AMPK) to promote the expression of proteins that function in TJs (28). Butyrate also regulates the gut microbiota by modulating the pH of the intestinal lumen (29), which is beneficial for bacteria that produce short-chain fatty acids (30);it maintains epithelial hypoxia status and limits overgrowth of nitrate-respiratory-dependent bacteria (31); and it stimulates the growth of villi and the production of mucin (32). Therefore, we suggest that the significant deficiency of *Faecalibacterium* in PBC patients contributed to the increased permeability of the intestinal mucosal barrier, and the migration of many bacteria into the liver *via* the gut-liver axis, ultimately manifesting as immune-mediated damage to the small bile ducts.

A third notable alteration is that the level of *Ruminococcaceae* was significantly reduced in PBC patients, also apparently unique to PBC patients from Zhejiang Province. *Ruminococcaceae* plays an important role in the conversion of primary bile acids into secondary bile acids (33), and impaired production of secondary bile acids can cause dysbiosis of gut microbiota and induce intestinal inflammation (34). Sinha et al. (35) compared a control group that had familial adenomatous polyposis (FAP) with a group that had ulcerative colitis, and showed severe depletion of *Ruminococcaceae* in the ulcerative colitis patients. Bajaj et al. (36) reported increased serum levels of IL-6 and lipopolysaccharide-binding protein and a decreased butyrate/isobutyrate ratio in patients with alcoholic liver disease, and that these changes were associated with a depletion of *Ruminococcaceae*. Therefore, we speculate that the depletion of *Ruminococcaceae* in PBC patients decreases the conversion of primary bile acids into secondary bile acids, leading to interruption of enterohepatic circulation and aggravation of cholestasis, and eventually to intestinal inflammation and exacerbation of the dysbiosis of gut microbiota.

The results of our KEGG functional annotation analysis suggested that the intestinal flora of PBC patients had abnormal expression of two pathways: lipid metabolism and biosynthesis of secondary metabolites. Many anaerobic intestinal microbes, such as species in the *Ruminococcaceae*, *Coprococcus*, and *Oscillospirales* (all detected in our study) produce short-chain fatty acids (SCFAs) by fermentation of dietary fiber (37, 38). SCFAs have a beneficial effect on health due to their anti-inflammatory effects (39), consistent with our finding of abnormal expression of this pathway in PBC patients. Secondary metabolite synthesis by the intestinal flora in our PBC group was also abnormal. This indicates insufficient conversion of primary bile acids into secondary bile acids in these patients, a condition that can further aggravate cholestasis (40). Dietary supplementation with *Ruminococcaceae* is a potential method for promoting bile acid metabolism and ameliorating cholestasis (41).

Our study has certain shortcomings, especially the relatively small sample size. However, we were only able to enroll 25 PBC patients because the incidence of this condition is very low, and the number of PBC patients who are treatment-naive is much smaller. Second, UDCA is relatively safe, and many patients with cholestatic liver disease have already taken UDCA before receiving a definitive diagnosis of PBC. However, because UDCA affects the composition of the intestinal flora, we had to exclude these patients. Third, a liver biopsy is not necessary for the diagnosis of PBC, but we excluded PBC patients who were diagnosed without liver biopsy results because of our need to use strict inclusion criteria and to enroll patients with definitive diagnoses.

Our study provided a comprehensive comparison of the gut microbiota of treatment-naive PBC patients and matched healthy controls from Zhejiang Province. The results provide new insights into the pathogenesis of PBC and the possible use of non-invasive biomarkers for the diagnosis or stratification of PBC patients.

## Data availability statement

The data presented in the study are deposited in the National Center for Biotechnology Information (NCBI) 16S rRNA database and the BioProject ID is PRJNA892581.

## Ethics statement

The studies involving human participants were reviewed and approved by Medical Ethics Committee of Hangzhou Xixi Hospital. The patients/participants provided their written informed consent to participate in this study.

## Author contributions

Study concept and design, acquisition of data, analysis and interpretation of data: Y-JZ, G-XY, S-LD. Drafting of the manuscript: Y-JZ, G-XY, BX. Critical revision of the manuscript for important intellectual content and study supervision: Q-FJ. All authors contributed to the article and approved the submitted version.
